# Study of the densification mechanisms of Al–Fe–Cr–Ti alloys during high-velocity compaction based on 3D MPFEM

**DOI:** 10.1038/s41598-026-62634-9

**Published:** 2026-07-24

**Authors:** Xianjie Yuan, Yuanpan Chen, Yirui Zhang, Xuanhui Qu, Haiqing Yin, Lijun Wang, Gang Li

**Affiliations:** 1 College of Mechanical Engineering, North China University of Water Resources and Electric Power, Zhengzhou, 450045 China; 2 Institute for Advanced Materials and Technology, University of Science and Technology Beijing, Beijing, 100083 China; 3 Collaborative Innovation Center of Steel Technology, University of Science and Technology Beijing, Beijing, 100083 China; 4 Beijing Key Laboratory of Materials Genome Engineering, Beijing, 100083 China; 5 College of Smart Manufacturing, Zhengzhou University of Economics and Business, Zhengzhou, 451191 China

**Keywords:** Al–Fe–Cr–Ti alloy, High-velocity compaction, 3D MPFEM, Impact energy per unit mass, Engineering, Materials science

## Abstract

Aluminum alloy materials are widely used in aerospace and related fields, among which Al–Fe–Cr–Ti alloys have attracted increasing attention owing to their low density and excellent comprehensive properties. However, the densification mechanisms of alloy powders during high-velocity compaction (HVC) remain insufficiently understood. In this study, a three-dimensional multi-particle finite element method (3D MPFEM) model was developed to simulate the HVC process of Al–Fe–Cr–Ti alloy powders and to evaluate the effects of friction coefficient *μ*, impact energy per unit mass *E*_*m*_, hammer mass *M*, and compaction velocity *v* on powder densification. The results show that increasing *μ* from 0.25 to 0.65 reduced kinetic-energy transfer and stress transmission, decreasing the relative density *ρ* of the green from 0.7076 to 0.6797. In contrast, increasing *E*_*m*_ from 55.58 to 144.67 J/g markedly improved densification, with the maximum relative density reaching 0.8881. Displacement-field analysis further revealed that appropriate combinations of *M* and *v* promote particle rearrangement and plastic deformation. Experimental validation confirmed that the simulated density evolution agreed well with the measured trend, although the predicted values were slightly lower. These findings indicate that 3D MPFEM can reasonably describe the macroscopic densification trend and provide qualitative particle-scale insights into deformation and energy-transfer behavior during HVC.

## Introduction

Aluminum alloys, owing to their exceptional strength, wear resistance, high-temperature stability, and corrosion resistance, exhibit tremendous application potential in aerospace, nuclear equipment, and advanced manufacturing sectors^1–3^. Among these, the Al–Fe–Cr–Ti system, as a significant class of lightweight alloys^4^, combines low density with excellent mechanical properties and oxidation resistance, making it a focal point of contemporary materials research.

In materials processing, powder metallurgy (PM)^5^ is widely employed for alloy production due to its near-net-shape forming, precise compositional control, and energy-efficient, environmentally friendly advantages. One of the most critical metrics in PM is the product density; thus, strategies to effectively enhance density and produce high-performance, cost-efficient powder metallurgy components have become central research objectives. In recent years, a range of advanced PM forming techniques has emerged, including high-velocity compaction^6^, atomized spray deposition^7^, injection molding^8^, hot pressing^9^, and die-wall lubrication technologies^10,11^.

High-velocity compaction (HVC) has become one of the research hotspots in the development of powder metallurgy technologies. It is a novel forming technique introduced by Höganäs AB, Sweden, in 2001, based on the HVC machine developed by Hydropulsor AB^12^. In this process, a hammer with a certain mass is driven by hydraulic or gravitational potential energy to impact the upper punch at high velocity, generating intense stress waves that are transmitted through the punch into the powder bed, thereby promoting powder densification. Jonsén et al.^13^ reported that, compared with conventional quasi-static pressing, high-velocity pressed metal powder greens exhibit improved compressibility, lower ejection force, and smoother sample surfaces. Skoglund et al.^14^ further found that HVC increased the density of certain iron-based powder materials by approximately 0.3 g/cm^3^ and improved both tensile strength and yield strength by about 20–25% compared with conventional compaction. However, despite these advantages, the dynamic densification mechanisms of alloy powders during HVC, especially the coupled effects of impact energy, hammer mass, compaction velocity, and friction, remain insufficiently understood.

In studies of granular material behavior, Essayah et al.^15^ investigated the relationship between contact force networks and energy dissipation in sand particle systems. Their findings indicate that over 70% of energy dissipation occurs within the weak contact network; however, the average energy dissipated per sliding contact in the strong contact network is higher, owing to the greater tangential contact forces. Wang et al.^16^ highlighted that the energy diffusion process in granular materials is influenced by inter-particle contact characteristics, primarily reflected in the distribution of contacts and the force transmission ratios. Yuan et al.^17^ investigated the energy dissipation characteristics of brittle particles. They reported that the particle breakage ratio significantly affects the energy dissipation rate, defined as the ratio of dissipated energy to input energy. As the particle breakage ratio increases, interparticle friction and collision become more intense, thereby increasing the energy dissipation rate.

During the HVC process, alloy powders exhibit complex physical behaviors, including inter-particle friction, localized plastic deformation, pore closure dynamics, and stress wave propagation. Numerous researchers have employed numerical methods to simulate and study these phenomena. Zhou et al.^18^, using 2D MPFEM combined with DEM, observed that post-compaction, the area of stress concentration at particle edges continuously increased as pore size decreased. Simultaneously, a small portion of stress concentration appeared on the longer side of the pores. Upon pressure release, significant residual stresses were detected in the contact zones between adjacent particles, with the maximum stress occurring at particle edges rather than within the interior regions. Li et al.^19^ employed the multi-particle finite element method (MPFEM) to conduct three-dimensional, particle-scale modeling of the compaction and densification behavior of soft-hard composite powders. Their study focused on the influence of initial packing structure and compaction pressure on both macroscopic and microscopic properties of the powder bed, with the developed model validated through physical experiments. Liu et al.^20^ utilized the FEM to analyze the densification mechanisms of iron-based powders during HVC, examining the relative density distribution under varying compaction velocities and impact energies. Their results indicated that regions of uneven density primarily occurred at the edges of the upper and lower surfaces of the green, and that higher compaction velocities led to a more uniform density distribution.

Beyond HVC, the 3D MPFEM approach has also been applied to other processes. Li et al.^21^ conducted a systematic study using 3D MPFEM on the MPRC process of tungsten-copper W-Cu mixed powders, focusing on particle-scale deformation, stress–strain distribution, and pore-filling behavior. They quantitatively assessed the effects of discharge voltage, copper content, and friction coefficient on densification, revealing that the incident stress waves generated by the pack layer, along with reflections from the mandrel, jointly influence the spatial distribution of compaction, resulting in pronounced radial density gradient.

Although MPFEM has been widely applied in powder forming simulations, its use in HVC remains limited because three-dimensional particle-scale modelling requires high computational cost. In particular, the effects of process parameters on Al–Fe–Cr–Ti alloy green quality have not been sufficiently clarified. Compared with previous MPFEM and DEM-FEM studies that mainly considered copper, W-Cu, iron-based, or other powder systems, the present work focuses on Al–Fe–Cr–Ti alloy powders and links three aspects within one 3D particle-scale framework: friction-dependent energy transmission, *E*_*m*_-controlled densification, and the non-equivalent effects of hammer mass and compaction velocity under the same *E*_*m*_. The aim is not to propose a new constitutive law, but to provide a quantitative and particle-scale interpretation that can support process selection for high-density Al–Fe–Cr–Ti alloy green.

## 3D MPFEM simulation method

### Definition of material properties

Yang et al.^22^, in studying the relationship between energy dissipation and the porosity of particle assemblies during HVC, found that under identical loading conditions, higher porosity in the particle assembly leads to greater energy dissipation. Therefore, the particle assemblies must be carefully designed, as will be discussed in "Establishment process of the 3D model" section.

Since the upper and lower punches and the die were treated as rigid bodies, only the deformable powder particles were assigned material properties. This study employed Al–Fe–Cr–Ti alloy powder. Pedrazzini et al.^23^ reported that Al–Fe–Cr–Ti alloys with nanoscale dispersed phases exhibit excellent mechanical properties and high room-temperature strength. The elastic modulus was set to 49 GPa according to the room-temperature compressive modulus reported for Al95Fe2Cr2Ti1 alloy by De Araujo et al.^24^. This value falls within the measured range of 44–51 GPa for different build orientations and was therefore adopted as a representative effective modulus for the present Al–Fe–Cr–Ti alloy system. The Poisson’s ratio was set to 0.33. The theoretical density was estimated using Eq. (1):

1 $$\rho _{{{\mathrm{TD}}}} = \frac{{\mathrm{n}}}{{\mathop \sum \nolimits_{{{\mathrm{i}} = 1}}^{{\mathrm{t}}} \left( {\frac{{{\mathrm{n}}_{{\mathrm{i}}} }}{{\rho _{{\mathrm{i}}} }}} \right)}}$$

Here, *ρ*_TD_ represents the theoretical density of the alloy, *ρ*_*i*_ is the theoretical density of element i, and *n*_*i*_ denotes the mass fraction of element i. The theoretical densities of Al, Fe, Cr, and Ti are listed in Table 1:

**Table 1:** Density of each element of Al, Fe, Cr, and Ti.

Element	Al	Fe	Cr	Ti
Density (g/cm^3^)	2.70	7.88	7.19	4.54

The theoretical density of the Al–Fe–Cr–Ti alloy, calculated using Eq. (1) and the densities of its constituent elements, is 2.92 g/cm^3^.

For large-deformation nonlinear problems, true stress–strain data are more suitable than engineering stress–strain data because changes in cross-sectional area and gauge length during deformation are considered. Engineering stress–strain curves are useful for preliminary material evaluation, but they may introduce error when plastic deformation is large.

Nikravesh et al.^25^ used an RVE-based continuum-atomistic multiscale technique to simulate heterogeneous nanoporous materials and employed true stress–strain curves to describe mechanical behavior. Because contact, ploughing, hardness, and nanoscale deformation differ from macroscopic behavior^26^, true stress–strain data are commonly used in constitutive modelling and finite element analysis.

The true stress and true strain are defined as follows:

2 $${{\sigma }}_{{{\mathrm{true}}}} {\text{ = }}\frac{{\mathrm{F}}}{{{\mathrm{A}}_{{\mathrm{0}}} }}$$

where *σ*_true_ is the true stress, *F* is the tensile force, and *A* is the current cross-sectional area of the specimen.

3 $$\varepsilon _{{{\mathrm{true}}}} = \ln \left( {\frac{{\mathrm{l}}}{{{\mathrm{l}}_{0} }}} \right)$$

where *ε*_true_ is the true strain, *l* is the current gauge length, and *l*_0_ is the original gauge length. The engineering stress–strain data were converted into true stress–strain data using Eqs. (4) and (5):

4 $$\sigma _{{{\mathrm{true}}}} = \sigma _{{{\mathrm{eng}}}} \left( {{\mathrm{1}} + \varepsilon _{{{\mathrm{eng}}}} } \right)$$

5 $$\varepsilon _{{{\mathrm{true}}}} = \ln \left( {1 + \varepsilon _{{{\mathrm{eng}}}} } \right)$$

where *σ*_true_ and *ε*_eng_ are the engineering stress and engineering strain, respectively.

**Fig. 1:**
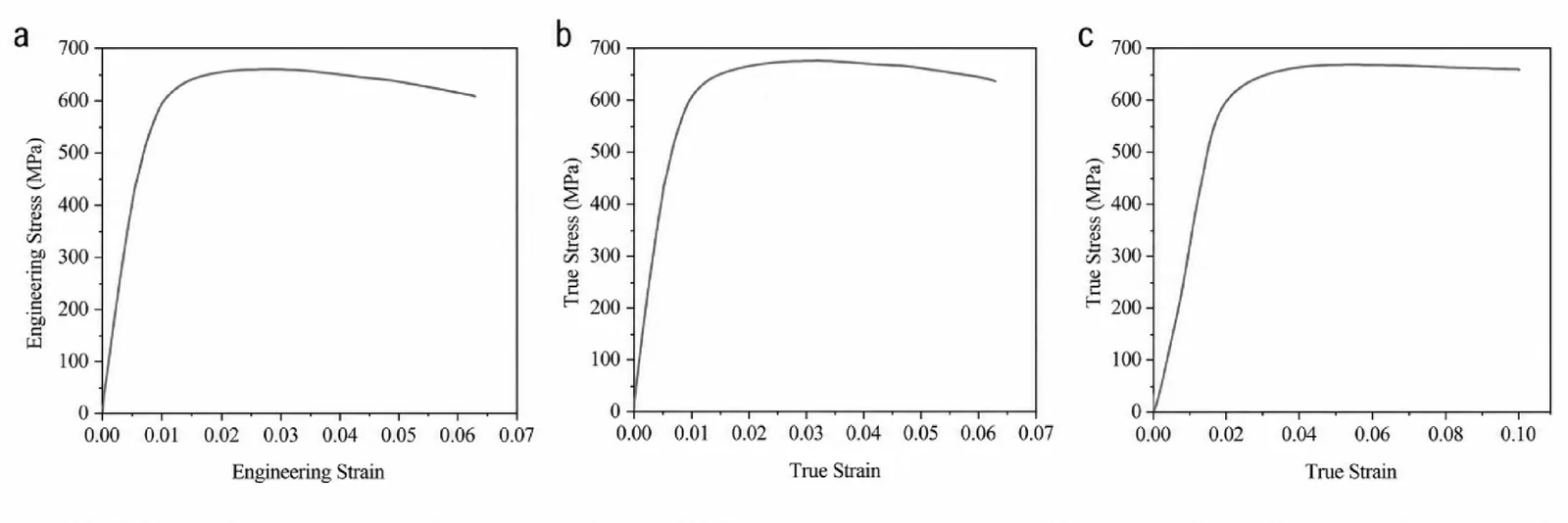
Stress–strain curves used for constitutive parameter fitting: (**a**) engineering stress–strain curve at room temperature and 1 × 10^−4^ s^−1^; (**b**) converted true stress–strain curve at room temperature and 1 × 10^−4^ s^−1^; (**c**) true stress–strain curve at room temperature and 1 × 10^−3^ s^−1^.

The engineering stress–strain curve of the Al–Fe–Cr–Ti alloy as shown in Fig. 1a reported by Pedrazzini et al.^23^, obtained under quasi-static tensile loading at room temperature and a strain rate of 1 × 10^−4^ s^−1^, was converted into the true stress–strain curve using Eqs. (4) and (5), as shown in Fig. 1b. The converted curve was then fitted to determine *A*, *B*, and *n* in the Johnson–Cook constitutive equation. The strain-rate sensitivity parameter *C* was estimated by jointly fitting the true stress–strain curves at 1 × 10^−4^ s^−1^ and 1 × 10^−3^ s^−1^, with the 1 × 10⁻³ s⁻¹ curve reported by H. Luo et al. ^27^, as shown in Fig. 1c.

### Determination of Johnson–Cook constitutive parameters

The Johnson–Cook constitutive model^28^ can describe the stress–strain evolution of materials during deformation, providing a theoretical basis for metal forming and serving as an important model in metal forming simulations. In finite element simulations of dynamic impact problems, the Johnson–Cook constitutive equation has been widely applied to the impact dynamics of metals and other materials^29–31^. Therefore, in this study, the Johnson–Cook constitutive equation is employed to describe the plastic large-deformation behavior and mechanical response during high-velocity compaction.

The Johnson–Cook constitutive model expresses flow stress as the product of strain hardening, strain-rate hardening, and thermal softening terms, as shown in Eq. (6).

Because the HVC simulations in this study were conducted at room temperature and temperature rise was not included, the thermal-softening term was omitted, leading to Eq. (7).

6 $$\sigma = \left( {{\mathrm{A}} + {\mathrm{B}}\varepsilon ^{{\mathrm{n}}} } \right)\left( {1 + {\mathrm{C}}\ln \frac{{\dot{\varepsilon }}}{{\dot{\varepsilon }_{0} }}~} \right)~\left( {1 - {\mathrm{DT}}^{{{\mathrm{*m}}}} } \right)$$

7 $$\sigma = \left( {{\mathrm{A}} + {\mathrm{B}}\varepsilon ^{{\mathrm{n}}} } \right)\left( {1 + {\mathrm{C}}\ln \frac{{\dot{\varepsilon }}}{{\dot{\varepsilon }_{0} }}~} \right)$$

where *σ* is the equivalent flow stress (MPa), *ε* is the equivalent plastic strain, ε̇ is the plastic strain rate, ε̇_0_ is the reference strain rate, and T* is the homologous temperature. *A* is the quasi-static yield stress (MPa), *B* is the strain-hardening coefficient (MPa), *C* is the strain-rate sensitivity coefficient, *m* is the thermal-softening exponent, and *n* is the strain-hardening exponent.

Room temperature and ε̇_0_ = 1 × 10^−4^ s^−1^ were used as the reference conditions. Under the reference strain rate, Eq. (7) can be simplified to Eq. (8). Pedrazzini et al.^23^ reported a yield strength of 544 MPa for the bulk Al–Fe–Cr–Ti alloy bar; therefore, *A* was set to 544 MPa. After *A* was determined, *B* and *n* were fitted from the true stress–strain curve.

**Fig. 2:**
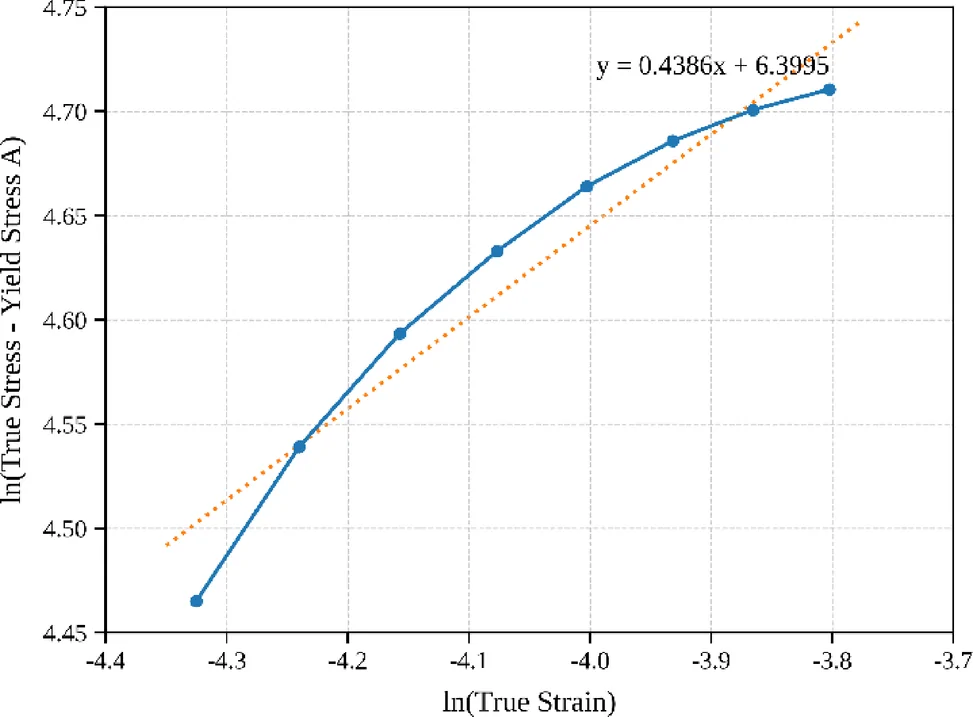
Linear regression of ln(*σ* − *A*) against ln(*ε*) used to determine the strain-hardening coefficient *B* and exponent *n*.

Rearranging Eq. (8) gives Eq. (9), and taking the natural logarithm of both sides gives Eq. (10). The intercept of the fitted line corresponds to ln *B* and the slope corresponds to *n*. The fitting results gave *B* = 601.845 MPa and *n* = 0.4386, as shown in Fig. 2.

8 $$\sigma = \left( {{\mathrm{A}} + {\mathrm{B}}\varepsilon ^{{\mathrm{n}}} } \right)$$

9 $$\begin{array}{*{20}c} {{\mathrm{s}} - {\text{A = B}} \cdot {\mathrm{e}}^{{\mathrm{n}}} } \\ \end{array}$$

10 $$\begin{array}{*{20}c} {\ln \left( {{\mathrm{s}} - {\mathrm{A}}} \right) = \ln \left( {\mathrm{B}} \right) + {\mathrm{n}} \cdot \ln \left( {\mathrm{e}} \right)} \\ \end{array}$$

**Fig. 3:**
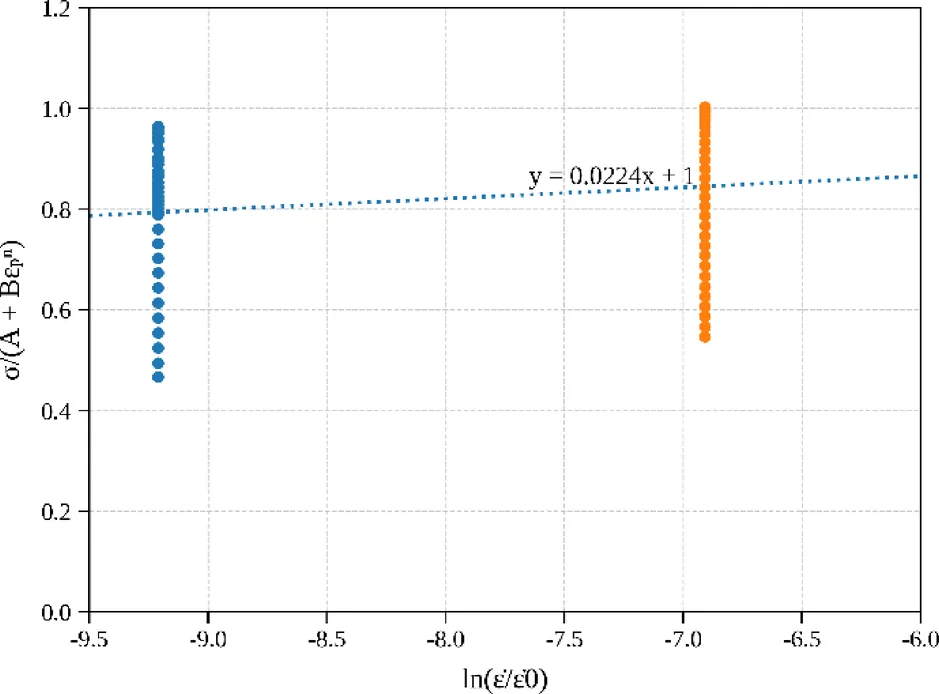
Regression used to estimate the Johnson–Cook strain-rate sensitivity coefficient *C* from the available stress–strain data.

The parameter *C* was estimated by fitting the true stress–strain curves of the Al–Fe–Cr–Ti alloy at room temperature under strain rates of 1 × 10⁻^4^ s⁻^1^ and 1 × 10⁻^3^ s⁻^1^. Equation (7) was rearranged as Eq. (11), and the fitted relationship shown in Fig. 3 gave *C* = 0.0224. Because *C* was obtained from low strain-rate data, its extrapolation to HVC conditions may introduce uncertainty in the predicted flow stress and densification response. Therefore, the simulated relative densities are used mainly to compare densification trends and relative differences among different process parameters, rather than to claim fully exact absolute predictions.

11 $$\frac{\sigma }{{{\mathrm{A}} + {\mathrm{B}}\varepsilon ^{{\mathrm{n}}} }} = 1 + {\mathrm{C}}\ln \left( {\frac{{\dot{\varepsilon }}}{{\dot{\varepsilon }_{0} }}~} \right)$$

The above data are summarized in Table 2.

**Table 2:** Parameters used for the HVC simulation of Al–Fe–Cr–Ti alloy powder.

Material parameter	Elastic modulus (GPa)	Density (g/cm^3^)	Poisson ratio	*A* (MPa)	*B* (MPa)	*C*	*n*
Value	49	2.92	0.33	544	601.845	0.0224	0.4386

After defining the material properties of Al–Fe–Cr–Ti for the HVC process, the required data were extracted from the corresponding frames in the Abaqus Visualization module and then imported into Excel for plotting. The final densification degree of the powder was determined from the ratio between the geometric volume occupied by powder particles in the final frame and the spatial volume after compaction. The relative density is expressed as Eq. (12):

12 $$\rho = \frac{{{\mathrm{V}}_{{\mathrm{p}}} }}{{\mathrm{V}}}$$

where *ρ* is the relative density, *V*_*p*_ is the volume of the powder particles after compaction, and *V* is the spatial volume after compaction.

### Establishment process of the 3D model

After the parameter settings in "Determination of Johnson–Cook constitutive parameters" section were completed, the model was established. The 3D MPFEM method is an integrated approach that combines the discrete element model with the finite element method. Owing to limitations in computational cost and simulation time, only a portion of the powder was ultimately selected for filling and compaction simulation in this study.

**Fig. 4:**
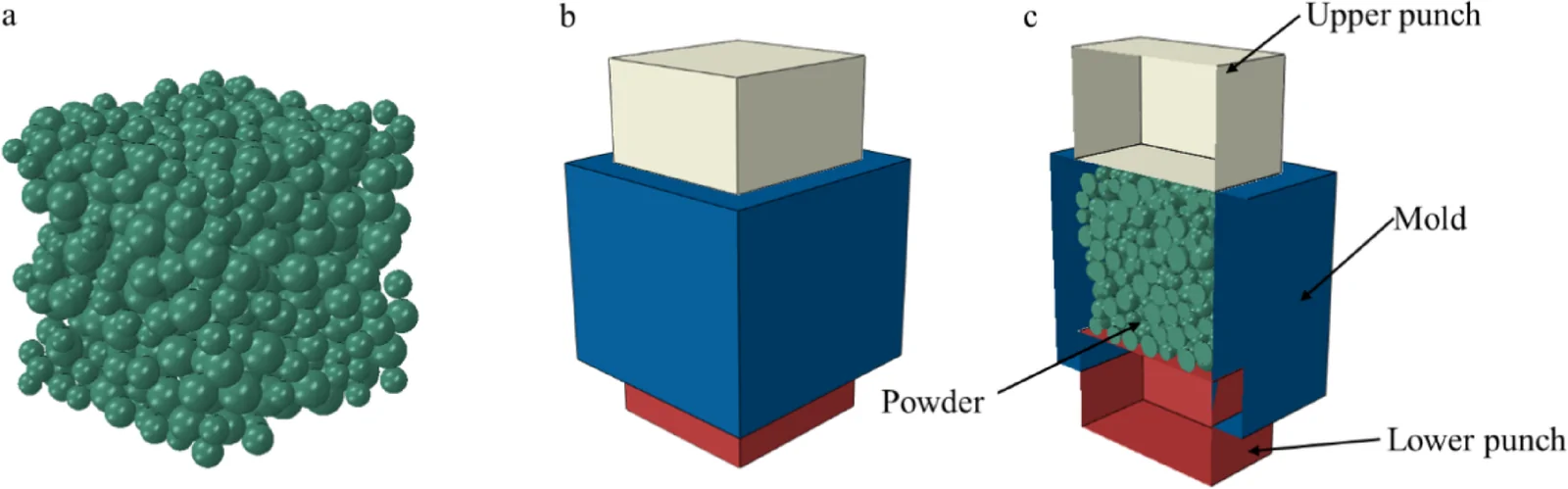
Three-dimensional modelling process: (**a**) powder-packing model; (**b**) assembled HVC model; (**c**) sectional view showing the upper punch, powder, mold and lower punch.

With the aid of CAD secondary development software, a 3D spherical powder model with different particle sizes was generated to represent the natural packing formed under gravity within a 0.5 mm × 0.5 mm × 0.5 mm spatial container. The particle size range was 38–70 *μ*m, as shown in Fig. 4a. This model was imported into Abaqus as a component for assembly. In Abaqus, the upper punch, lower punch, and die were modeled separately, and the final assembly was completed in the Abaqus/Assembly module. Figure 4b shows the assembled model, while Fig. 4c presents its sectional view.

When establishing the high-velocity compaction simulation model of metal powder in Abaqus, the powder particles are defined as deformable bodies and meshed using tetrahedral elements, while the upper punch, lower punch, and die are defined as rigid bodies and meshed using hexahedral elements. Since the motion and loading of rigid components are primarily controlled through reference points, reference points are assigned to the upper punch, lower punch, and die, respectively.

To simulate the falling impact of the hammer, the corresponding mass and initial velocity were applied to the reference point of the upper punch. Constraint boundary conditions were imposed on the reference points of the lower punch and die. Considering the instantaneous impact, large deformation, and strong nonlinear characteristics of HVC, the explicit dynamic method based on the central difference scheme was adopted. The total simulation time was set to 0.1 s to include both the main impact stage and the subsequent decay of kinetic energy; the main densification response occurred within approximately the first 10 ms.

The interactions among the model components are defined using surface-to-surface contact. The tangential contact behavior is described by the penalty friction model, while the normal contact behavior is defined using the hard contact model. To simplify the analysis, the friction coefficients for particle–particle and particle–rigid body interactions are uniformly assigned the same value.

The present 3D MPFEM model was established to represent a local particle-packing region rather than a full-size die. To ensure comparability among different cases, the same particle-size distribution, packing procedure, meshing strategy, contact formulation, and time-increment control were used in all simulations. Since an explicit dynamic solver was adopted, the calculation does not involve iterative convergence in the same manner as an implicit analysis. However, mesh density, stable time increment, initial packing, and contact parameters may still affect the predicted stress field, particle deformation, and relative density. In this work, the model was validated mainly by comparing the simulated and experimentally measured green relative densities. Therefore, the relative density results are used to evaluate the overall densification trend, whereas the stress and displacement contours are interpreted as qualitative indicators of force transmission and particle rearrangement.

## Results and discussion

### Effect of varying friction coefficients

During HVC, friction within the powder and between the powder and the die is considered a key factor influencing the relative density of green. Khoei et al.^32^ demonstrated, using the molecular dynamics (MD) method, that die-wall friction significantly affects both the mechanical behavior of nanopowders during compaction and the mechanical properties of the final components. Numerical simulations further reveal that die-wall friction alters the pressure distribution profile and reduces the uniformity of nanoparticle deformation. Zhang et al.^33^, combining particulate mechanics theory with DEM, found that increasing the friction coefficients at the sidewalls and between particles generally amplifies the heterogeneity of microscopic contact forces, with interparticle friction exerting a more pronounced effect than sidewall friction.

Therefore, this study investigated the effect of the friction coefficient on the HVC response. The friction coefficient was varied as *μ* = 0.25, 0.45, and 0.65, while the geometric model, boundary conditions, material properties, hammer mass (*M* = 15 kg), and compaction velocity (*v* = 35 mm/s) were kept constant. Each case was submitted as an independent Dynamic, Explicit analysis with a total simulation time of 0.1 s. The bottom reaction force, kinetic energy, and green relative density were extracted for comparison.

**Fig. 5:**
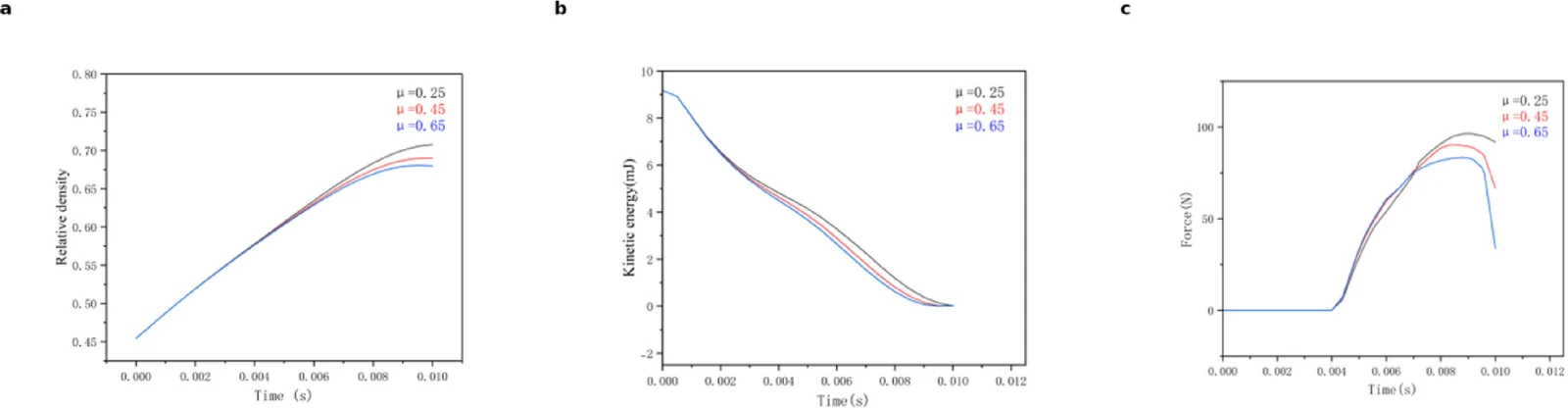
Effect of friction coefficient on HVC response: (**a**) Evolution of green relative density; (**b**) Kinetic-energy history; (**c**) Bottom reaction force. The hammer mass and compaction velocity were fixed at *M* = 15 kg and *v* = 35 mm/s.

As shown in Fig. 5a, increasing the friction coefficient reduced the relative density of the green from 0.7076 at *μ* = 0.25 to 0.6897 at *μ* = 0.45 and 0.6797 at *μ* = 0.65. The kinetic-energy histories in Fig. 5b show that the three cases were similar at the beginning of compaction, whereas from approximately 2–9 ms the kinetic energy for *μ* = 0.25 remained higher than that for *μ* = 0.45 and *μ* = 0.65. This difference is attributed to frictional energy dissipation between particles and between particles and the die wall. The bottom reaction force in Fig. 5c also decreased as *μ* increased, indicating that higher friction weakened stress-wave transmission through the powder bed.

### Effect of hammer mass and compaction velocity

During the HVC powder compaction process, the free-fall motion of the hammer, upon contact with the upper punch, facilitates energy transfer. In this study, the impact energy provided by the hammer is expressed by Eq. (13). However, Yan et al.^34^, in their investigation of Ti powder densification during HVC, found that *E*_*m*_ more reasonably characterizes the densification process. This is defined by Eq. (14), with Eqs. (15) and (16) derived through mathematical calculations.

13 $$\begin{array}{c}E=\frac{1}{2}M{\mathrm{v}}^{2}\end{array}$$

14 $$\begin{array}{c}{E}_{m}=\frac{M{v}^{2}}{2m}\end{array}$$

15 $$\begin{array}{c}v=\sqrt{\frac{2m{E}_{m}}{M}}\end{array}$$

16 $$\begin{array}{c}M=\frac{2m{E}_{m}}{{v}^{2}}\end{array}$$

where *E* is the impact energy of the hammer, *E*_*m*_ is the impact energy per unit mass of compacted powder, *M* is the hammer mass, *v* is the compaction velocity of the hammer, and *m* is the mass of the compacted powder.

Zhou et al.^18^ and Yan et al.^34^, through both experimental and simulation studies on high-velocity compaction, demonstrated that increasing *E*_*m*_ effectively enhances the relative density of green. From the aforementioned equations, it is evident that *E*_*m*_ is closely related to both hammer mass and compaction velocity and can be increased either by raising the compaction velocity or by augmenting the hammer mass.

**Fig. 6:**
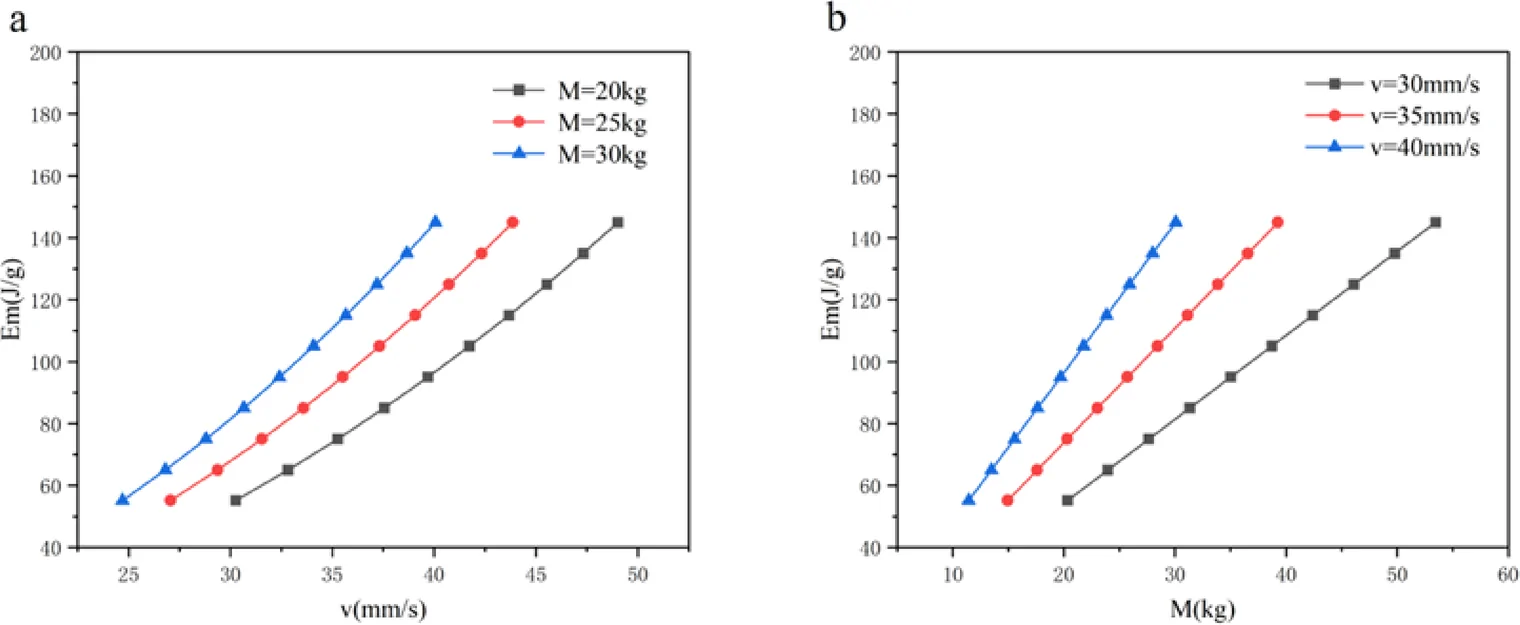
Relationship between impact energy per unit mass and process parameters: (**a**) compaction velocities corresponding to selected hammer masses; (**b**) hammer masses corresponding to selected compaction velocities.

**Fig. 7:**
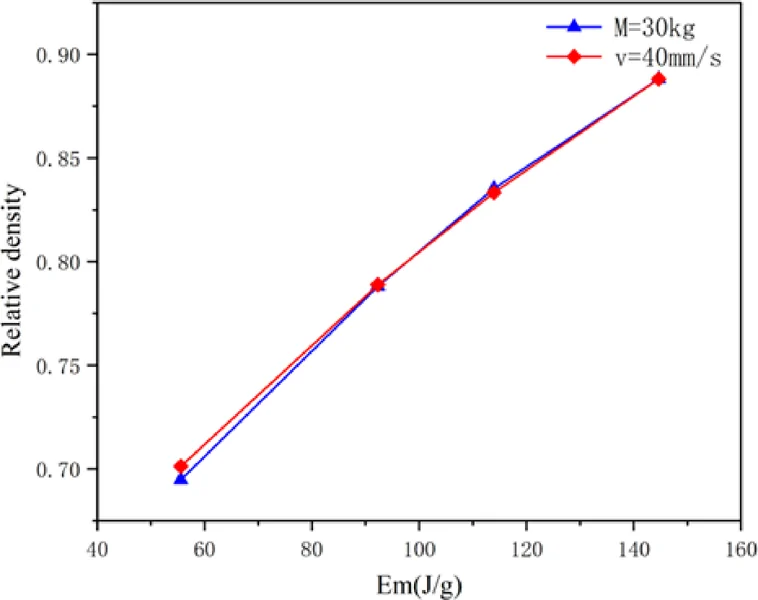
Relative density as a function of impact energy per unit mass when either hammer mass or compaction velocity was fixed.

As shown in Fig. 6, *E*_*m*_ increases with increasing compaction velocity at a given hammer mass and also increases with increasing hammer mass at a given compaction velocity. Therefore, the same target *E*_*m*_ can be obtained through different combinations of *M* and *v*. In practical processing, increasing the compaction velocity is usually the more direct way to increase *E*_*m*_. As shown in Fig. 7, within *E*_*m*_ = 55.58–144.67 J/g, when *M* was fixed at 30 kg and *v* increased from 24.75 to 40 mm/s, the relative density increased from 0.6947 to 0.8881. When *v* was fixed at 40 mm/s and *M* increased from 11.48 to 30 kg, the relative density increased from 0.7013 to 0.8881. The variation trend of relative density with increasing *E*_*m*_ is consistent with previous HVC experiments on Al–Fe–Cr–Ti alloys^35^.

When *E*_*m*_ was 55.58, 92.3, and 144.67 J/g, five different combinations of hammer mass *M* and compaction velocity *v* corresponded to each energy level, as shown in Table 3. At the two lower energy levels, the maximum relative densities were 0.7079 and 0.7932, with variances of *ρ* of 2.34 × 10^−5^ and 2.67 × 10^−5^, respectively. At *E*_*m*_ = 144.67 J/g, the maximum relative density increased to 0.8881, while the variance of *ρ* increased to 1.89 × 10^−4^. This higher variance indicates that the density result becomes more sensitive to the selected *M*-*v* combination at higher *E*_*m*_.

**Table 3:** Hammer masses, compaction velocities, relative density, and variance of *ρ* at *E*_*m*_ = 55.58, 92.3, and 144.67 J/g.

*E*_*m*_ (J/g)	*M* (kg)	*v* (mm/s)	*ρ*	Variance of *ρ*
55.58	11.48	40	0.7013	2.34 × 10^−5^
	15	35	0.7076	
	20	30	0.7079	
	25	27.11	0.7028	
	30	24.75	0.6947	
92.3	15	45.18	0.7794	2.67 × 10^−5^
	19.14	40	0.7889	
	25	35	0.7932	
	30	31.95	0.7882	
	34.03	30	0.7810	
144.67	20	48.99	0.8798	1.89 × 10^−4^
	25	43.82	0.8804	
	30	40	0.8881	
	39.18	35	0.8839	
	53.34	30	0.8495	

Combined with the analysis in Fig. 7, these results show that increasing *E*_*m*_ allows the powder bed to receive more effective impact energy, resulting in more complete pore closure and more pronounced plastic deformation. However, the higher-energy cases also show greater dispersion in relative density, indicating that hammer mass *M* and compaction velocity *v* are not fully interchangeable even when *E*_*m*_ is kept constant. Therefore, the *M*-*v* combination should be optimized rather than selected solely from the energy value.

In summary, based on the analysis of Table 3, the final relative density of the green during high-velocity compaction is jointly influenced by *M* and *v*, and a non-monotonic optimization range exists. Specifically, when *E*_*m*_ remains constant, different combinations of *M* and *v* can produce different densification outcomes. Once either *M* or *v* exceeds a certain threshold, further increasing the mass or velocity may instead reduce the final relative density of the green. Therefore, it is necessary to determine the optimal combination of *M* and *v* that maximizes the green relative density.

Taking *E*_*m*_ = 55.58 J/g as an example, when the hammer mass increased from 20 to 30 kg, the relative density decreased from 0.7079 to 0.6947. Similarly, when the compaction velocity increased from 30 to 40 mm/s under the same *E*_*m*_, the relative density decreased from 0.7079 to 0.7013. Thus, at *E*_*m*_ = 55.58 J/g, the optimal parameter combination was *M* = 20 kg and *v* = 30 mm/s. At *E*_*m*_ = 92.3 J/g and *E*_*m*_ = 144.67 J/g, the maximum relative densities were 0.7932 and 0.8881, corresponding to *M* = 25 kg, *v* = 35 mm/s and *M* = 30 kg, *v* = 40 mm/s, respectively. In "Analysis of particle states during HVC" section, the combinations of *M* and *v* are further examined from a particle-scale displacement perspective.

### Analysis of particle states during HVC

This section analyzes particle deformation during the high-velocity compaction of powder particles, building on the findings presented in "Effect of varying friction coefficients" and "Effect of hammer mass and compaction velocity" sections.

The friction coefficients for particle–particle and particle–die-wall interactions are kept homogeneous. The hammer mass *M* is fixed at 15 kg, and the compaction velocity is set to 35 mm/s, ensuring that *E*_*m*_ remains constant. A controlled-variable method is then employed to independently investigate the particle deformation behavior under different values of *μ*.

**Fig. 8:**
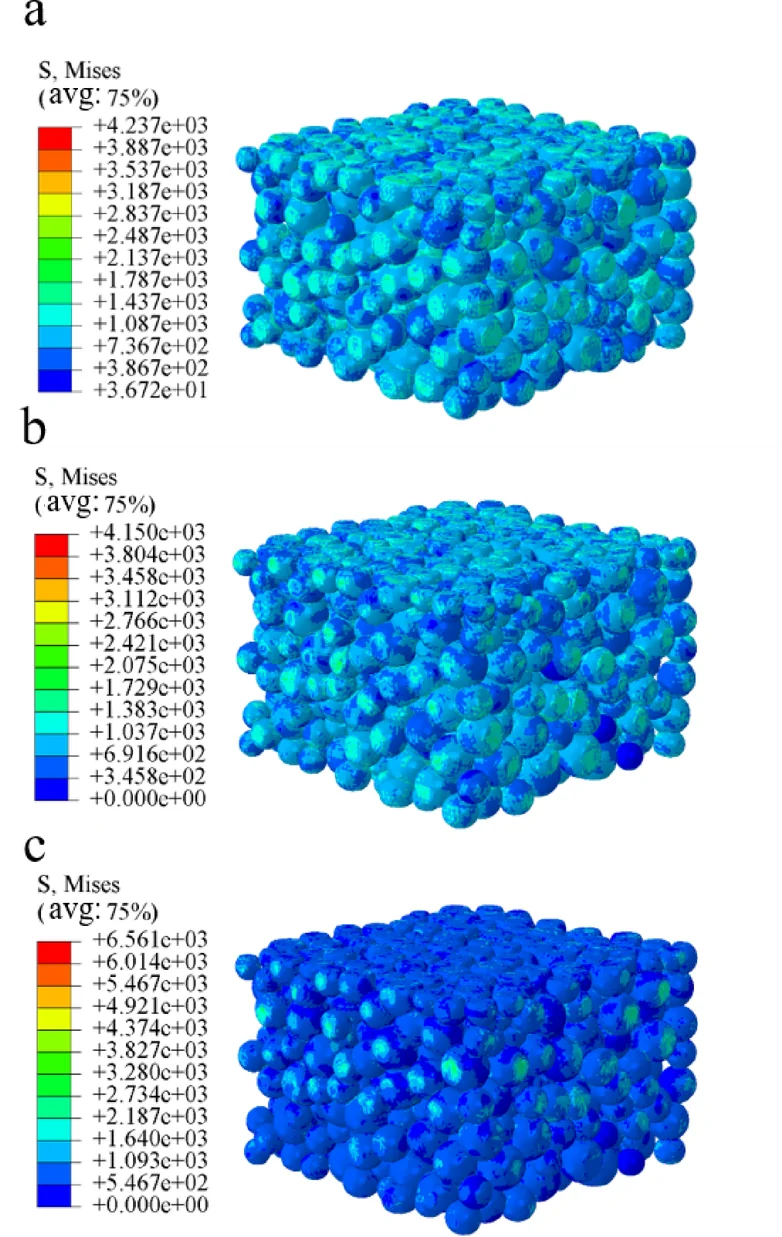
Mises stress contour plots after final compaction under different friction coefficients: (**a**) *μ* = 0.25; (**b**) *μ* = 0.45; (**c**) *μ* = 0.65.

Figure 8 presents the Mises stress contours at the final compaction stage for *μ* = 0.25, 0.45, and 0.65 under the same *E*_*m*_ and observation plane. The low-friction cases show more continuous stress transfer through the particle assembly, whereas the high-friction case contains a larger proportion of low-stress regions. This behaviour is consistent with the kinetic-energy and bottom-force results in Fig. 5, suggesting that friction dissipates part of the input energy and reduces axial stress-wave transmission.

Because the particle-scale stress field was not directly measured experimentally, the stress contours are interpreted as qualitative indicators of force transmission rather than as independently validated local stress values. The key validated quantity remains the macroscopic relative density, which is compared with experimental measurements in "Experimental verification" section.

**Fig. 9:**
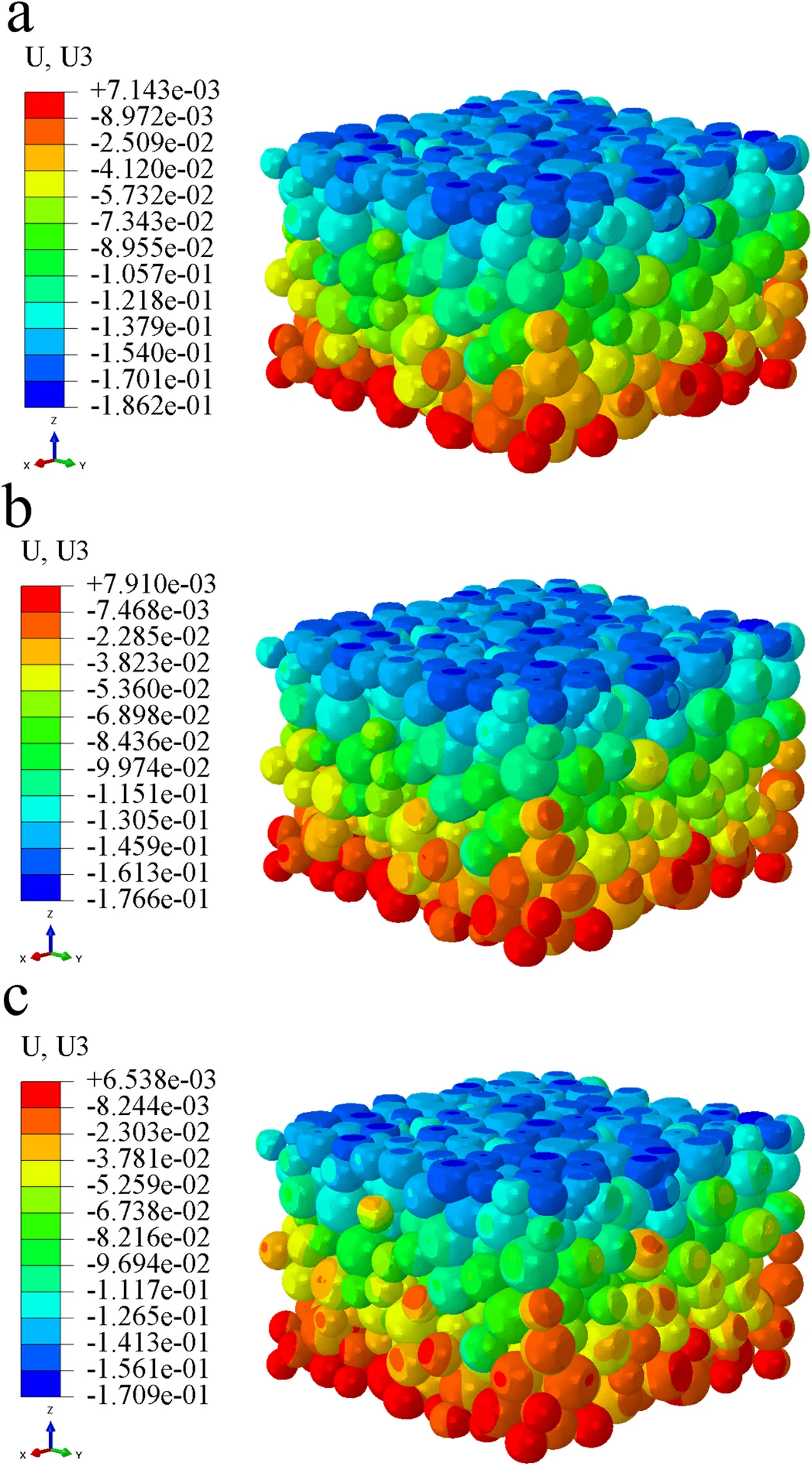
Z-axis displacement contour plots after final compaction under different friction coefficients: (**a**) *μ* = 0.25; (**b**) *μ* = 0.45; (**c**) *μ* = 0.65.

As shown in Fig. 9, the Z-axis displacement contours under different friction coefficients exhibit a similar downward compaction pattern, but the displacement magnitude decreases as *μ* increases. This trend is consistent with the density results in "Effect of varying friction coefficients" section, indicating that higher friction suppresses particle rearrangement and reduces pore filling under the same *E*_*m*_.

The maximum displacement of the green continuously decreases as the friction coefficient increases. As discussed in "Effect of varying friction coefficients" section, the relative density of the green decreases from 0.7076 at *μ* = 0.25 to 0.6897 at *μ* = 0.45, and further to 0.6797 at *μ* = 0.65. This indicates that the relative density also decreases with increasing friction coefficient. Therefore, when *E*_*m*_ remains constant, the variation trend of the maximum displacement of the green under different friction conditions is consistent with that of the relative density.

A similar compaction behavior was also reported by Chen et al.^36^. In that study, the green was analyzed under conventional compaction and under different ultrasonic amplitudes. The results showed that, as the ultrasonic amplitude increased, both the relative density and the maximum displacement of the green increased accordingly.

**Fig. 10:**
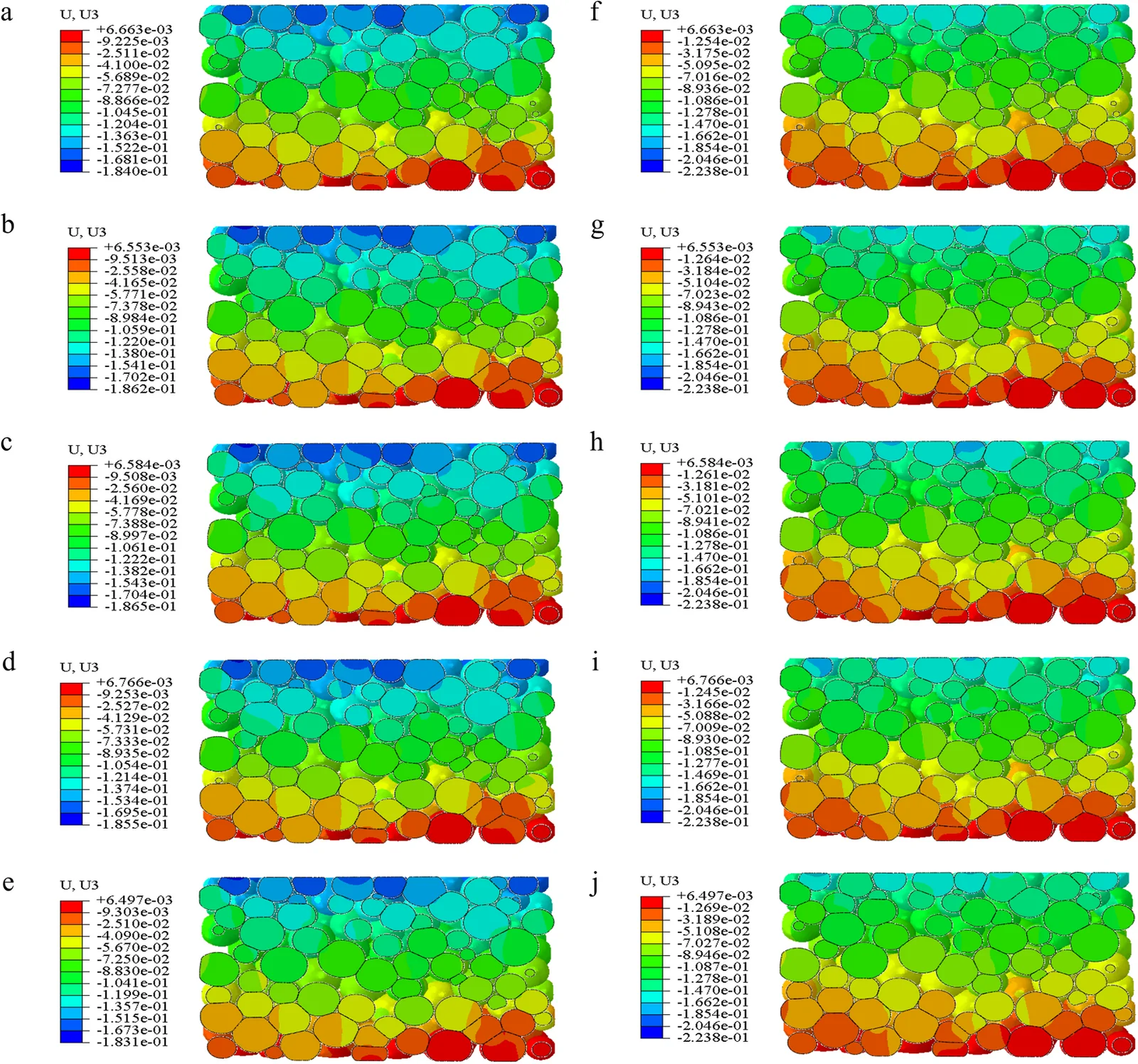
Z-axis displacement contour plots of the x = 0.15 mm cross-section under different *M*-*v* combinations at *E*_*m*_ = 55.58 J/g. Panels (a-e) show actual displacement scales, and panels (f-j) use a fixed colour scale for comparison: (**a**, f) *M* = 11.48 kg, *v* = 40 mm/s; (**b**, **g**) *M* = 15 kg, *v* = 35 mm/s; (**c**, **h**) *M* = 20 kg, *v* = 30 mm/s; (**d**, **i**) *M* = 25 kg, *v* = 27.11 mm/s; (**e**, **j**) *M* = 30 kg, *v* = 24.75 mm/s.

The green was sectioned along the plane x = 0.15 mm, and the Z-axis displacement of this cross-section was examined. Under *μ* = 0.25 and *E*_*m*_ = 55.58 J/g, the displacement contour plots corresponding to different *M*-*v* combinations are shown in Fig. 10. The left column shows the actual displacement scale for each case, whereas the right column uses a fixed scale to facilitate direct visual comparison.

According to Table 3 and Fig. 10, the five parameter combinations at *E*_*m*_ = 55.58 J/g are: (a) *M* = 11.48 kg, *v* = 40 mm/s; (b) *M* = 15 kg, *v* = 35 mm/s; (c) *M* = 20 kg, *v* = 30 mm/s; (d) *M* = 25 kg, *v* = 27.11 mm/s; and (e) *M* = 30 kg, *v* = 24.75 mm/s. The corresponding relative densities are 0.7013, 0.7076, 0.7079, 0.7028, and 0.6947, respectively.

The maximum Z-axis displacement magnitudes for these five cases are approximately 0.1840, 0.1862, 0.1865, 0.1855, and 0.1831 mm, respectively. Both relative density and maximum displacement first increase from case (a) to case (c) and then decrease from case (c) to case (e), indicating that the *M* = 20 kg, *v* = 30 mm/s combination provides the most favourable densification at *E*_*m*_ = 55.58 J/g.

Fixed color scale limits were used to compare particle velocity variations, following Liu et al. ^37^. In the fixed-scale contours on the right side of Fig. 10, the darkest colour corresponds to the largest preset displacement magnitude. The broader high-displacement region in case (h) further supports the conclusion that the *M* = 20 kg and *v* = 30 mm/s combination promotes more effective particle rearrangement under this energy level.

Overall, the displacement results show that the maximum particle displacement is positively related to the final green relative density under the examined conditions. Low friction promotes particle rearrangement and stress transmission, whereas excessive friction increases energy dissipation. Under a constant *E*_*m*_, the *M*-*v* combination also affects the displacement field, confirming that impact energy per unit mass alone cannot fully determine the densification state.

### Experimental verification

This section builds upon previous experimental studies of high-velocity compaction of Al–Fe–Cr–Ti powders^35^. Under single-stroke conditions, the variation of green density *ρ*_*g*_ with compaction energy *E* was measured and converted into the variation of relative density with *E*_*m*_. The experimental relative density was calculated as the ratio of green density to theoretical density.

**Fig. 11:**
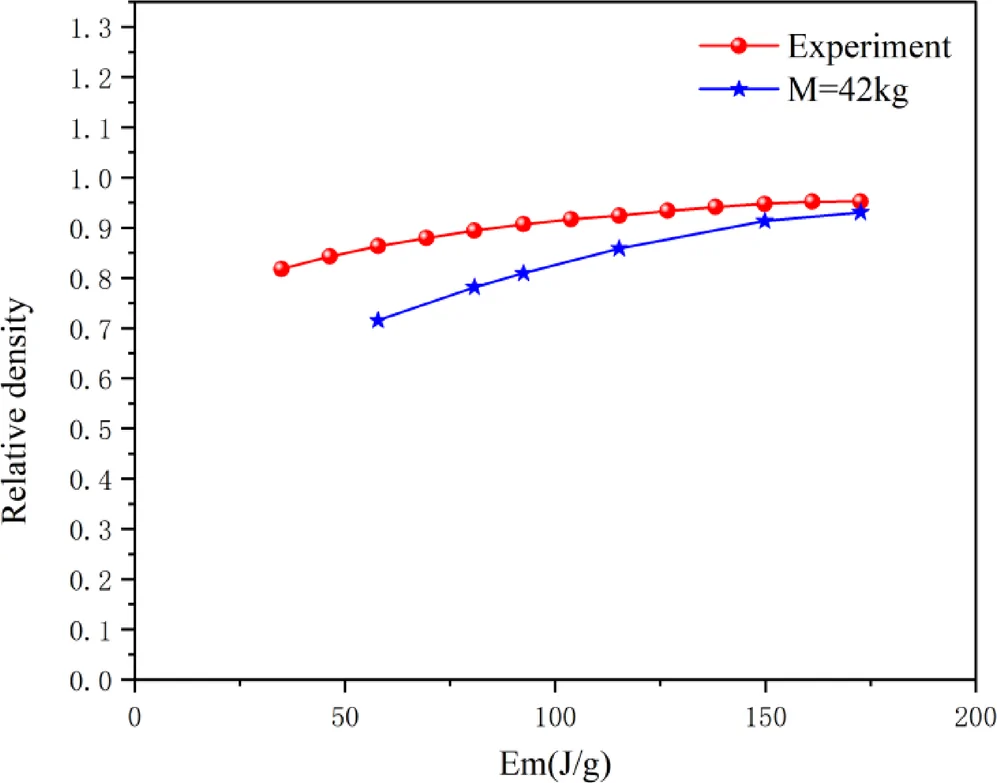
Relative density of the green under high-velocity compaction: comparison between experimental and simulation results.

With the hammer mass held constant at 42 kg, *E*_*m*_ was adjusted by varying the compaction velocity. As shown in Fig. 11, when *E*_*m*_ increased from 34.9 to 172.61 J/g, the experimental relative density increased from 0.8180 to 0.9525. The simulation results within *E*_*m*_ = 57.85–172.61 J/g also showed a monotonic increase, with the relative density rising from 0.7156 to 0.9307. The simulated densities were slightly lower than the experimental values, which may be related to simplified powder constitutive behaviour, contact assumptions, friction treatment, and energy dissipation in the numerical model.

Physically, powder densification involves conversion of impact energy into particle rearrangement, plastic deformation, and pore closure. At low *E*_*m*_, densification is dominated by particle rearrangement and elastic deformation, causing rapid pore closure. At higher *E*_*m*_, most large pores have already been reduced and plastic deformation becomes dominant, so the increase in relative density becomes more gradual. The agreement in the rapid-then-gradual trend indicates that the 3D MPFEM model captures the main macroscopic densification tendency, although particle-scale stress and displacement results should still be treated as qualitative mechanistic evidence.

## Conclusions

The high-velocity compaction process of Al–Fe–Cr–Ti alloy powder was numerically simulated using the 3D MPFEM method and compared with experimental density measurements. The results show that this method can reasonably describe the main densification trend, energy-transfer behaviour, and particle-scale deformation characteristics during HVC. Because the present study relies mainly on numerical simulation and macroscopic density validation, the local stress and displacement fields should be interpreted as qualitative mechanistic indicators. Further high-strain-rate material tests, mesh-independence studies, and particle-scale validation would strengthen the general applicability of the proposed mechanism.

This study mainly investigated the effects of friction coefficient, impact energy per unit mass *E*_*m*_, and the combination of hammer mass and compaction velocity on the relative density of Al–Fe–Cr–Ti alloy green compacts. The main conclusions are as follows:

1. The friction coefficient significantly affects green densification. As *μ* increases, kinetic energy decreases and force transmission weakens. When *μ* increases from 0.25 to 0.65, the relative density decreases from 0.7076 to 0.6797, indicating that frictional resistance suppresses particle rearrangement and plastic deformation.
2. Increasing either compaction velocity or hammer mass increases *E*_*m*_. However, under the same *E*_*m*_, different *M*-*v* combinations still produce different relative densities. The variances of *ρ* at *E*_*m*_ = 55.58, 92.3, and 144.67 J/g were 2.34 × 10^−5^, 2.67 × 10^−5^, and 1.89 × 10^−4^, respectively. The larger variance at 144.67 J/g indicates that the *M*-*v* combination should be optimized under high-energy conditions. The highest relative density in this study is 0.8881 at *M* = 30 kg and *v* = 40 mm/s.
3. Particle rearrangement can be inferred qualitatively from the maximum displacement of the green. Lower friction produces larger particle displacement in the upper and middle regions, leading to more complete rearrangement and pore filling. At *E*_*m*_ = 55.58 J/g, the *M* = 20 kg and *v* = 30 mm/s combination gives the largest displacement and the highest relative density among the five combinations examined at that energy level.
4. Experimental density measurements validate the applicability of the 3D MPFEM model at the macroscopic level. Both the experimental and simulation results show that relative density increases with *E*_*m*_ and follows a rapid-then-gradual growth pattern. This agreement indicates that 3D MPFEM is useful for analysing and optimizing HVC process parameters, while further validation is needed for quantitative particle-scale stress and displacement fields.

## Data Availability

The numerical data used to generate the figures and tables are included in this article or are available from the corresponding author upon reasonable request. Representative Abaqus input files, output data, and post-processing procedures can be provided to support reproducibility of the reported simulations.
